# A Network Approach to the Study of the Dynamics of Risk Spillover in China’s Bond Market

**DOI:** 10.3390/e23070920

**Published:** 2021-07-20

**Authors:** Zhewen Liao, Hongli Zhang, Kun Guo, Ning Wu

**Affiliations:** 1 School of Economics and Management, University of Chinese Academy of Sciences, Beijing 100190, China; liaozhewen16@mails.ucas.ac.cn (Z.L.); hlzhang0101@gmail.com (H.Z.); wuning20@mails.ucas.ac.cn (N.W.); 2 Key Laboratory of Big Data Mining and Knowledge Management, Chinese Academy of Sciences, Beijing 100190, China; 3 Research Center on Fictitious Economy & Data Science, Chinese Academy of Sciences, Beijing 100190, China; 4 National Science Library, Chinese Academy of Sciences, Beijing 100190, China

**Keywords:** bond market, fixed income security, risk spillovers, structural entropy, generalized variance decomposition, complex network

## Abstract

Since 2018, the bond market has surpassed the stock market, becoming the biggest investment area in China’s security market, and the systemic risks of China’s bond market are of non-negligible importance. Based on daily interest rate data of representative bond categories, this study conducted a dynamic analysis based on generalized vector autoregressive volatility spillover variance decomposition, constructed a complex network, and adopted the minimum spanning tree method to clarify and analyze the risk propagation path between different bond types. It is found that the importance of each bond type is positively correlated with liquidity, transaction volume, and credit rating, and the inter-bank market is the most important market in the entire bond market, while interest rate bonds, bank bonds and urban investment bonds are important varieties with great systemic importance. In addition, the long-term trend of the dynamic spillover index of China’s bond market falls in line with the pace of the interest rate adjustments. To hold the bottom line of preventing financial systemic risks of China’s bond market, standard management, strict supervision, and timely regulation of the bond markets are required, and the structural entropy, as a useful indicator, also should be used in the risk management and monitoring.

## 1. Introduction

From 2007 onwards, the Subprime Crisis brought about drastic changes in the global economic and financial system, exposing a series of loopholes in traditional financial institutions and regulatory systems, as well as showing a rising trend of cross-country risk contagion overtime [1]. Because of the down-speed shifting of economic development with a new normal medium speed after decades of high-speed and extensive growth, China’s government now emphasizes quality of economic development and views financial risk management as a more important consideration than ever before.

Despite the outbreak and spread of the COVID-19 pandemic, the decoupling of the global economy, and the rise of populism having had a major impact on the global economy, China’s domestic economy has endeavored to deepen supply-side structural reforms, which give full play to China’s ultra-large-scale market advantages and domestic demand potential, and build new development that promotes both domestic and international cycles, as well as keeping China’s economy energetic. At this stage of the pattern, China’s financial system has entered a new period full of volatility and uncertainty after the long-term accumulation of systemic risks. In keeping with the findings from the research of Fang et al. [2], with the increasing openness of the Chinese economy, Chinese financial markets are becoming more integrated with those of developed markets, and Chinese financial markets are demonstrating a growing impact on global financial markets over time, especially during periods of turbulence.

Macroeconomic variables often suffer from structural changes due to changes in institutional reforms, policies, crises, and other factors [3], while systematic macroeconomic risks often tend to accumulate in the form of bubbles silently [4], only bursting with the outbreak of a crisis. When the bubble bursts, the spillover effect among institutions involved in the economic activities would become significant, expanding the range of the damage, so the systematic risks caused could not be ignored. So, understanding the risk contagion mechanism of the shocks in the financial market is significantly helpful, as well as crucial for investors for the purposes of asset allocation, asset pricing, risk management, and arbitrage trading. Generally, the investors who face asset price fluctuation, including both institute investors and individual investors, mostly use negatively correlated assets to complete their asset allocation, minimizing the portfolio risks. However, few investment institutions realize that to avoid systemic risks from being transmitted to themselves, identifying systemic risks and related systemically important institutions is a crucial step. Obviously, it is essential for regulators and governments to understand the transmission mechanism of financial shocks, since extreme volatility shock spillover causes financial unpredictability and brings about unexpected market impacts. In order to stabilize the price fluctuation in financial markets, ensuring that it is in a better condition to serve the real economy, the policymaker should develop appropriate policies to prevent large market impacts of volatility shocks from extreme events [5].

**Figure 1:**
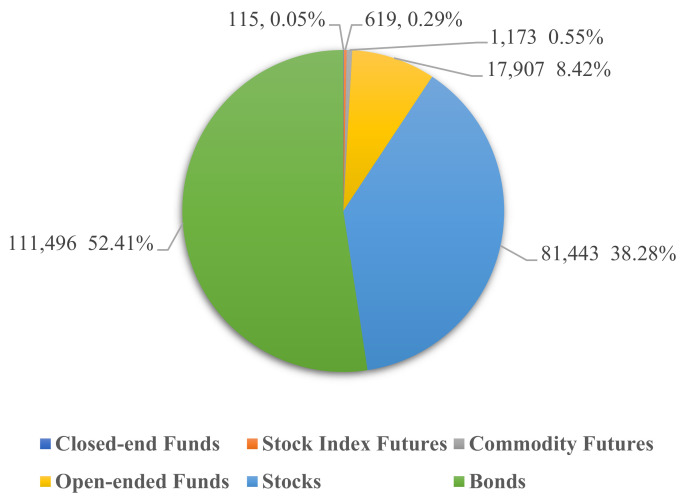
The scale of China’s security market, as of 28 October 2020 (Unit: RMB billion).

As Figure 1 shows, up until 28 October 2020, China’s bond market has a tremendous scale of RMB 112 trillion, accounting for 52.41% of China’s entire securities market. According to this fast-growing and tremendous volume, the bond market is almost the biggest investment area in China, only second to the real estate market. Although there are few individual investors in the bond market, the importance of the prosperity and stability of China’s bond market cannot be emphasized too much, as well as the significance of controlling the volatility and risk of this market.

However, few studies have focused on the inner dynamics of China’s bond market. In our research, we innovatively combine the methodology of complex networks and the traditional econometric method, and instead of using the indices data of the financial market, we pioneeringly use the interest rate data of different bond types in China’s bond market, which provide a better representation of the inner factors of China’s bond market in a relatively micro view, instead of traditional quarterly data from the balance sheets of financial institutes.

There are 23 types of bonds included in our research. Using the traditional econometric model will face the problem of degrees of freedom caused by too many variables, so the complex network method will be more appropriate. In this paper, the bond market is regarded as a complex system that includes different types of bonds as nodes. The spillover indexes among the exchange rate fluctuations are used to construct the network. To make the relationship among bonds more intuitive and clearer, and to show the most effective path in the risk contagions process, the minimum spanning tree (MST) method is applied to analyze the influence structure. Thus, the key nodes and the key path of volatility risk contagion can be detected. This paper is organized as follows: the first section introduces the background to our research; the data are briefly introduced in Section 2; the techniques of network analysis and the results are discussed in Section 3; and finally, we end with a conclusion in Section 4.

## 2. Literature Review

The current economic crisis illustrates a critical need for new and fundamental understanding of the structure and dynamics of economic networks. Economic systems are increasingly built on interdependencies, implemented through trans-national credit and investment networks, trade relations, or supply chains that have proven difficult to predict and control [6]. For investigation of the risk in financial markets, various methods have been used in related research, and the simulation approach is often used, especially when a financial network is involved. Battiston and Caldarelli [7] used the simulation approach and stress tests to focus on the role of linkages within the two dimensions of contagion and liquidity, and to examine the mechanism of the contagions of systemic risk in financial networks, and they found that with respect to the issue of the determination of systemically important financial institutions, the findings indicate that both from the point of view of contagion and from the point of view of liquidity provision, there is more to systemic importance than just size. Ponta and Cincotti [8] presented and studied an information-based multi-asset artificial stock market characterized by different types of stocks and populated by heterogeneous agents to determine the influences of agents’ networks on the market’s structure. They concluded that the network is necessary in order to achieve the ability to reproduce the main stylized facts, but also that the market has some characteristics that are independent of the network and depend on the finiteness of traders’ wealth.

The research on financial market contagion or spillover effects has been widely studied in the economic and management aspects, and is also used in our research for its robustness and interpretability. For example, Diebold and Yilmaz [9] proposed several connectedness measures built from pieces of variance decompositions, and they argued that they provide natural and insightful measure connectedness among financial asset returns and volatilities by using directed networks to make the relationship more clear. Su [10] used the MHS-EGARCH model, finding that there are negative return and volatility spillover effects between currency and stock markets, and the stock indices in emerging markets have a higher return and a higher risk. Dey and Sampath [11] analyzed spillovers in returns and volatility among five major financial assets in India, especially the shock from the USA, by using a generalized vector autoregressive model, and they find that banking, real estate and gold matter the most for India. There are a number of similar studies such as Georgiadis [12], Yang and Zhou [13], and Miranda-Agrippino and Rey [14] that show that the US monetary policy could cause a considerable spillover impact in the global financial market. Morana and Bagliano [15] analyzed business cycle spillovers and synchronization within groups of old and new European Union countries and found out that spillovers are beneficial for the common monetary policy of the European Union. Lyocsa et al. [16] studied the connectedness of a sample of 40 stock markets across five continents using daily dosing prices and return spillovers based on Granger causality by building a complex network of the global stock market. In conclusion, they found that the probability of return spillover from a given stock market to other markets increases with market volatility and market size and decreases with higher foreign exchange volatility.

In addition, closing hours are important for information propagation. The research of Tsai and I-Chun [17] is interesting, as they used the data of economic policy uncertainty (EPU) in four countries or regions, finding that EPU in China is the most influential, and its contagion risk spreads to different regional markets, except for Europe; the effect of EPU in the United States is inferior to that in China; EPU in Japan merely influences contagion risk in emerging markets; contagion risk in European markets is not influenced by the four EPU indices; and EPU in Europe is not influenced by contagion risk in the global stock market. Huo and Ahmed [18] examined the impact of the Shanghai-Hong Kong Stock Connect by using the BEKK GARCH model. They found that the new Stock Connect does contribute to the increasing importance of the Chinese mainland stock market and economic activity, and found a leading role of the Shanghai stock market in the Hong Kong stock market in terms of both mean and volatility spillover effects after the Stock Connect. Narayan et al. [19] examined the relationship between stock returns and mutual fund flows in India by applying a generalized VAR model. In addition, it was also found that the spillover index defined in their research could be used to predict stock returns and mutual fund flows. Mensi et al. [20] studied the linkages both within and between stock and foreign exchange (FX) markets via three higher moments of return distributions (volatility, skewness and kurtosis), finding that cross-asset market linkages are of a similar magnitude to intra-asset-market linkages within emerging market, but the latter are stronger in developed markets. Christiansen [21] used a GARCH volatility–spillover model to analyze the volatility spillover from the US and aggregate European bond markets into individual European bond markets, and the weekly data of multiple bond indices issued by JPMorgan were used in his research. In his conclusion, strong statistical evidence of volatility spillover from the US and aggregate European bond markets was found. Additionally, it is interesting to find that the bond markets of EMU countries became much more integrated after the Euro was first issued, and this was mainly driven by the convergence in interest rates under the unified monetary policy [22], documented asymmetry in return and volatility spillover between equity and bond markets in Australia for daily returns during the period 1992–2006 by using a bivariate GARCH modelling approach. The illuminative result from their research is that negative bond market returns spillover into lower stock market returns, whereas good news originating in the equity market leads to lower bond returns, and the spillover effects are stronger in a one-way channel from the bond market to the equity market.

There are plenty of studies using matrices and network methods to study financial markets. Junior and Franca used the eigenvalues and eigenvectors of the correlations matrices of some of the main financial market indices in the world, showing that the high volatility of markets is directly linked with strong correlations between them, and their conclusion provided a good explanation of the major financial market crises that occurred between 1987 and 2008 [23]. Matesanz’s team analyzed co-movements in a wide group of commodity prices during the time period 1992–2010. Their methodological approach was based on the correlation matrix and the networks inside. Through this approach, they were able to summarize global interaction and interdependence, capturing the existing heterogeneity in the degrees of synchronization between commodity prices. Their results suggest that speculation and uncertainty are drivers of the sharp slump in commodity prices’ synchronization [24]. There are also several studies on the inter-market spillover effect in China, such as the research of Zhu et al. [25], or about inter-bank spillover effect, such as the research of Bao, Wu and Li [26].

It is worth mentioning that the hybrid methods such as structural entropy have gradually become more commonly used in financial research: Murialdo and Ponta [27] presented a perspective on the intangible complexity of economic and social systems by investigating the dynamical processes producing, storing and transmitting information in financial time series by using the moving average cluster entropy approach. Shi et al. [28] used gray relational analysis and empirical mode decomposition to decompose and reconstruct the sequences to obtain the evolution trend and periodic fluctuation of systemic risk, and used structural entropy as a measurement to verify the results, showing that the systemic risk of China’s stock market as a whole shows a downward trend, and the periodic fluctuation of systemic risk has a long-term equilibrium relationship with the abnormal fluctuation of the stock market. Bielik [29] used entropy combined with technical indicators of the stock market, such as MACD, to find predictable market parts and improve the automated and non-automated trading strategies in the financial market.

Except for the methods mentioned above, the rise of econophysics, a fundamentally new approach in finance, suggests that the influence between the two disciplines has become less unilateral than in the past. Jovanovic’s research aimed at analyzing the unexpected influence of financial economics on physics. With this purpose, their study went one step further in the dialogue between econophysics and economics. Indeed, by investigating the reciprocal influence between the two fields, their paper identified some areas for a better cross-fertilization between the fields [30]. Kutner’s research presented some of the achievements of econophysics and sociophysics which appear to us to be the most significant [31], and Schinckus’ study aimed at analyzing how econophysicists implicitly promote a Duhemian way of perceiving scientific research by expanding their work into economics [32].

## 3. Methodology

### 3.1. Generalized Vector Autoregressive Forecast Error Variance Decomposition

To measure the risk spillover effect of the complex network of bond markets, we calculated the volatility spillover indices based on a generalized VAR in which the FEVD is invariant to the variable ordering initially proposed by Francis et al. [33] and Diebold and Yilmaz [34]. The details are shown as follows:

At the very first beginning, it is necessary to establish a VAR model with N variables in the lagging P period with stable covariance:

(1) $$x_{t}=\overset{p}{\underset{i=1}{\sum }}\phi_{i}x_{t-i}+{Ò}_{t}$$

where $x_{t}=\left(x_{1t},x_{2t},\cdots ,x_{Nt}\right)$ is a vector with *N* endogenous variables, $\phi_{i},\;i=1,2,\cdots ,p$ is a *N*-dimensional autoregressive coefficient matrix, the mean of the error vector ${Ò}_{t}$ is zero, and the covariance matrix is denoted as Σ. When the VAR model is stationary, the (1) could be convert to a moving average formula:

(2) $$x_{t}=\overset{\infty }{\underset{j=0}{\sum }}A_{j}{Ò}_{t-j}$$

$A_{i}$ should meet the condition that $A_{i}=\phi_{1}A_{i-1}+\phi_{2}A_{i-2}+\cdots \phi_{n}A_{i-n}$, and $A_{0}$ is a *N*-dimensional unit matrix, and when *j* < 0, $A_{i}=0$.

Secondly, in order to measure the spillover effect between variables and the total spillover effect, this study defines the spillover effect between variables: the spillover effect of variable $x_{j}$ on variable $x_{i}$ is defined as the variance of the *H*-step prediction error of $x_{i}$ that is impacted by the $x_{j}$ part where *i* ≠ *j*. The *H*-step represents the time span of the forecast error of the VAR model—that is, the number of periods of variance decomposition, which can be represented by Formula (3):

(3) $$\theta_{ij}^{H}=\frac{\sigma_{jj}^{-1}\sum_{h=0}^{H-1}{\left(e_{i}^{\prime }A_{h}\sum e_{j}\right)}^{2}}{\sum_{h=0}^{H-1}\left(e_{i}^{\prime }A_{h}\sum A_{h}^{\prime }e_{i}\right)}$$

While $\sigma_{ij}^{-1}$ is the standard deviation form of the prediction error of the *j*th variable, $e_{i}$ is a *N* × 1 vector, where the *i*th element is 1, and the rest are zero. $\theta_{ij}^{H}$ represents the spillover effect of variable $x_{j}$ on variable $x_{i}$, with it being noted that $\sum_{j=1}^{N}\theta_{ij}^{H}$, so $\theta_{ij}^{H}$ should be standardized:

(4) $${\hat{\theta }}_{ij}^{\tilde{H}}=\frac{\theta_{ij}^{H}}{\sum_{j=1}^{N}\theta_{ij}^{H}}$$

and now $\overset{N}{\underset{j=1}{\sum }}\theta_{ij}^{H}=1$, $\overset{N}{\underset{i,j=1}{\sum }}\theta_{ij}^{H}=N$. The matrix $\theta^{H}=\left[{\tilde{\theta }}_{ij}^{H}\right]$ shows the spillover effect among *N* variables, and the main diagonal element represents the overflow effect of the variable itself, while the non-diagonal element represents the overflow effect between different variables.

The percentage form of the total spillover effect can be obtained from Formula (4):

(5) $$TS=\frac{\sum_{i,j=1,i\neq j}^{N}\theta_{ij}^{\tilde{H}}}{\sum_{i,j=1}^{N}\theta_{ij}^{\tilde{H}}}\times 100=\frac{\sum_{i,j=1,i\neq j}^{N}\theta_{ij}^{\tilde{H}}}{N}\times 100$$

Regarding the total spillover index *TS*, add the non-diagonal elements in the resulting matrix $\theta^{\tilde{H}}=\theta_{ij}^{\tilde{H}}$ as the numerator of the total spillover index, and the denominator of the total spillover index is obtained by adding up all the elements in the matrix. In this way, the total spillover effect index measures the degree of the total spillover effect between in bond markets, so it can be used as a quantitative indicator to measure the degree of bond market correlation, as well as its risk of spreading. The bigger the spillover index is, the greater the volatility of the bond market due to the risk spillovers between different bond varieties will be, which in turn shows that the links between financial markets are very close.

### 3.2. The Complex Network, the MST Method and Structural Entropy

A complex network generally comprises several nodes and edges linking them. The node is the basic unit of a complex network, which is the abstract expression of an “individual” in the real world [35]. The edge is an expression of the relationship between the units and could be given weight accordingly to describe the extent of the relationships quantitatively [36]. In human social activities, the most common complex network is the small world network [37]; while talking about the Internet, scholars of complex networks usually define it as a scale-free network [38]. Different types of complex networks usually have different characteristics of their edges and nodes [39], and here in our research, $w_{ij}$ represents the weight of the edge linking node *i* and node *j*, where *i* = 1, 2, 3, …, n, *j* = 1, 2, 3, …, n, where n is the amount of nodes in a certain network. For an undirected network,

(6) $$w_{ij}=w_{ji}$$

The research also uses the weighted degree to represent the importance of nodes, which is defined as:

(7) $$dw_{i}=\underset{j\in v\left(i\right)}{\sum }w_{ij}$$

where $v_{\left(i\right)}$ is the set of nodes linking to node *i*. The stronger the degree of correlation with other nodes is, the more important the node is.

In our study, the spillover index of 1st difference to the interest rate data has been used, shown as:

(8) $$w_{ij}={\left(spillover\;index\right)}_{i\;to\;j}$$

It should be noted that $w_{ij}$ here represents the weight of the edge from *i* to *j* in a directed network, and the ${\left(spillover\;index\right)}_{i\;to\;j}$ here could be calculated from $\theta_{ij}^{H}$ in Formula (3), and vice versa.

To detect a clearer structure of the complex network of bond market, we apply the minimum spanning tree (short as MST) method [40] that has been previously applied to this research aspect [41,42]. This method selects the indices with the closest interactions among all the indices and generates a visual presentation of the relationship with n − 1 edges in the tree. When using the MST method, the relatively insignificant edges are discarded and there is only one route between any two nodes, which means that the complex network constructed by the MST shows more concise and clearer risk contagion relationships in China’s bond market, and that it is easier to discern the key bond types in the risk spillover complex network.

To construct the MST, the spillover index firstly needs to be converted into a “distance” coefficient as the input of the Kruskal algorithm. Following these references [28,43], we use nonlinear mapping:

(9) $$d_{ij}=\sqrt{2\left(1-{\left(spillover\;index\right)}_{ij}\right)}$$

to obtain the distance $d_{ij}$, noting that $d_{ij}\;$=$\;d_{ji}$ in the undirected graph, and ${\left(spillover\;index\right)}_{ij}$ could be defined as:

(10) $${\left(spillover\;index\right)}_{ij}=\left({\left(spillover\;index\right)}_{i\;to\;j}+{\left(spillover\;index\right)}_{j\;to\;i}\right)/2$$

and ${\left(spillover\;index\right)}_{i\;to\;j}$ here represents the spillover index from node *i* to node *j*, and vice versa. It should be pointed out that the index here represents the percentage of the spillover of node *i* to node *j* to the total impact of *j* by the volatility spillover. The Kruskal algorithm [44] is used in this paper to construct the MST complex network.

$d_{ij}\;$represents the “distance” coefficient, which should be used as input of the Kruskal algorithm to generate an MST complex network. In an MST complex network, the relatively insignificant edges are discarded and there is only one route between any two nodes, and the weights of the edges are inversely proportional to $d_{ij}$.

In addition, for a better vision to observe the network’s dynamics, the network’s structural entropy was calculated in this study, which is often used as a quantitative measurement of the complexity of the complex network system [45]. Generally, a non-fully connected network structural entropy $E_{degree}$ could be calculated as follows:

(11) $$E_{degree}=-\overset{N}{\underset{i=1}{\sum }}p_{i}\log p_{i}$$

where *N* is the total number of the nodes in the complex network, and $p_{i}$ in (11) could be calculated by the degree of node *i*, just as follows:

(12) $$P_{i}=\frac{degree\left(i\right)}{\sum_{i=1}^{N}degree\left(i\right)}$$

After the complex network has been constructed, some useful indicators can be used to analyze the characteristics of the network, such as degree and centrality. For node *i* in the complex network, the degree of node *i* represents the number of its neighboring nodes. Compared with the node’s degree, the centrality is a relatively complicated indicator type, which is usually used to measure the node’s relationship with the other nodes in some aspect. In this research, three kinds of centrality are mentioned: closeness centrality, betweenness centrality and eigenvector centrality [46].

Closeness centrality is an indicator that the higher the closeness centrality a node has, the closer the distance from the node to other node in the complex network, and vice versa [47]. The closeness centrality $C_{v}$ could be calculated as follows:

(13) $$C_{v}=\frac{V-1}{\sum_{i\neq v}^{N}d_{vi}}$$

where $d_{vi}$ represents the shortest distance from node *v* to node *i*, and V is the total number of nodes.

The betweenness centrality is usually used to measure the node’s central significance to a complex network; the greater the number of shortest paths passing through a node, the higher its betweenness centrality [48]. The formula of calculating betweenness centrality of node *i*, which is denoted as $B_{i}$, is as follows:

(14) $$B_{i}=\frac{SP_{i}}{SP_{total}}$$

where $SP_{i}$ represents the number of the shortest paths passing through node *i*, while $SP_{total}\;$stands for the total number of the shortest paths in the complex network.

The eigenvector centrality, shortened to eigen centrality, is an indicator often used to measure the number and the importance of its neighboring nodes [49]. The most famous algorithm used in search engine, called PageRank, is one kind of eigenvector centrality. The greater the number of nodes and the more important neighboring nodes the node has, the higher the eigen centrality of the node has, and the highest eigen centrality in the complex network is set as 1 by normalization. For a given graph G with *v* number of nodes, let $A=\;\left(a_{v}t\right)$ be the adjacency matrix, and the eigen centrality ${\mathrm{EC}}_{i}$ of node *i* can be defined as [50]:

(15) $${\mathrm{EC}}_{i}=x_{v}=\frac{1}{\lambda }\underset{t\in M\left(v\right)}{\sum }x_{t}=\frac{1}{\lambda }\underset{t\in G}{\sum }a_{v,t}x_{t}$$

where $M\left(v\right)$ is a set of the neighbors of *v* and *λ* is a constant. With a small rearrangement, this can be rewritten in vector notation as the eigenvector equation:

(16) Ax=λx

## 4. Data Description

**Figure 2:**
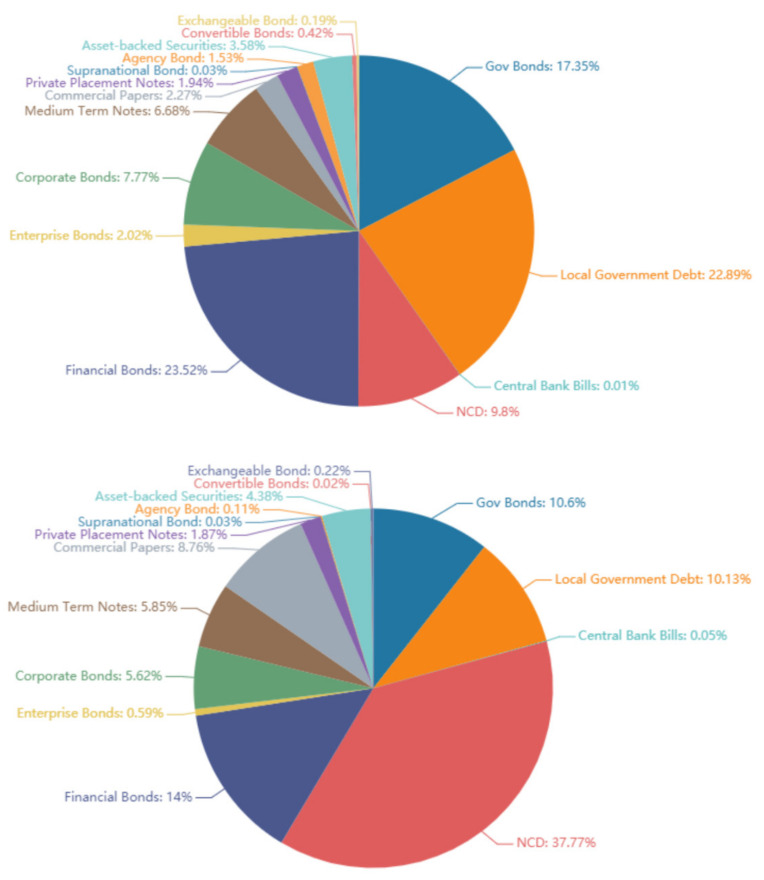
The percentage of the remaining size of different bond types in China’s bond market (all maturities), the upper figure shows bonds with all maturity, the lower figure shows bonds with 1 year of remaining maturity; as of 28 October 2020.

The primary goal of this study is to provide a historical narrative on the dynamics of risk spillover networks of China’s bond market. For this purpose, data preprocessing of this research is shown as follows:

At the very beginning of our research, proper data type and bond maturity should be chosen. We first studied the size and liquidity of different bond type to obtain a holistic view of China’s bond market today. After obtaining data from WIND, we present the data in Figure 2.

To take the multiple fundamental elements of bonds into consideration, and in order to control some factors to concentrate on the evolution of risk and volatility spillover in the network of bond markets, 23 types of bond interest rate data were chosen, including the credit spread of SOE, R007, DR007, and SHIBOR, as the benchmark interest rate data. In addition, in order to obtain a good representation of the results, and avoiding the potential price distortion of low credit rating bonds, this study mainly focuses on relatively high credit rating bonds in China, with a rating higher than AA (AA included) generally. For the same reason, this study mainly chooses the bonds which have 1 year of remaining maturity, because these bonds could reflect the features of both the monetary market and the capital market. The time interval is from 15 December 2014 to 28 October 2020, which is the time interval that guarantees that the above interest rate data could be obtained, and it is believed that the daily data, which cover almost last 6 years, ensure a good performance and representation. As Table 1 shows, all the data are stationary or stationary after the first difference, and the generalized vector autoregressive volatility spillover variance decomposition model is based on the first differenced data.

To take the several fundamental elements of bonds into consideration, and in order to control some factors, such as credit rating and term structure, to concentrate on the evolution of risk and volatility spillover network of bond markets as mentioned before, the descriptive statistical analysis of first differenced data are as follows in Table 2, where the *t*-statistics come from the Dickey–Fuller unit root test (AIC):

It can be clearly seen from Table 2 that all the data are stationary. In the same scale, the mean values of the data are all near zero and are all less than zero, which is mainly because the risk-free rate had a declining trend in this period, which could be indicated by the mean value of TREASURY and CDB.

## 5. Empirical Results

### 5.1. Static Spillover Effect Analysis

By using the model mentioned in Section 2, firstly, the full-sample spillover index is based on the FEV decomposition 12 days in advance. Each variable is related to the sequence of daily changes in bonds’ interest rates. Therefore, the measurement of the diagonal element *i* = *j* is the spillover effect within a certain type of bond, while the non-diagonal element (*i* ≠ *j*) captures the spillover effect between different bond categories, and the last line is the acquisition of each variable Additionally, the total spillover effect passed.

As is shown in Table 3, it can be concluded that:

For bond varieties with a high liquidity and large trading volume, such as financial bonds, government bonds, short- and medium-term notes and other mainstream varieties traded in the inter-bank bond market, the volatility spillover effects of these varieties are significantly higher. The volatility of the entire bond market overflows the complex network of greater systematic importance, mainly because the price of these bonds has become the benchmark of similar bonds to some extent.

For the same types of bonds with the same maturity, the sub-categories with high credit ratings have higher spillover effects, greater system importance, and a deeper influence on the system, compared with the low-credit rating bonds. This might be attributed to the high-credit rating bonds having better liquidity and the risk aversion of investors, and, furthermore, there may be some internal regulation and guidance in investment institutions that mean that the trader could only buy bonds which have a credit rating of AAA or AA+, which might enhance these effects. This can be clearly seen from related corporate bonds, medium- and short-term notes, and urban investment bonds as to their total static spillover effect, where the high-rating bonds have a bigger contribution to others than low-rating bonds.

**Figure 3:**
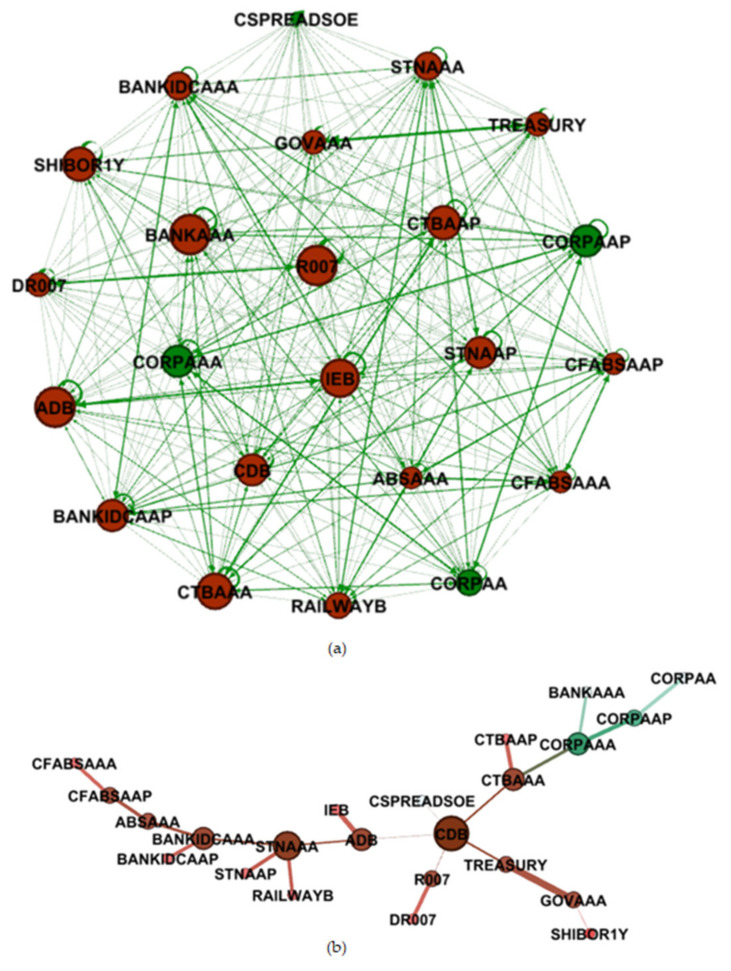
The complex network of bond markets constructed by the static spillover index of the full sample: (**a**) is the fully connected graph, (**b**) is the MST graph (the nodes which represent bond varieties traded in the inter-bank market are colored as red, noting that self-loops here mean the spillover effect from historical data of itself).

For the purpose of a better illustration of the result of the study regarding the mechanisms of the complex network of risk spillovers in China’s bond market, we used the static spillover index to construct the relevant complex network and used Gephi to draw Figure 3a as follows, noting that the size of nodes corresponds with the importance of the bond: the bigger the node is, more important the bond is in the complex network. The thickness of the link between the nodes indicates the strength of the influence of one bond on the other bond, in the direction of the linkage.

It can be seen from the complex network diagram in Figure 3a that as a result of their large trading volume (accounting for nearly 90% of the entire bond trading volume), good liquidity, and relatively fairer pricing, meaning they are preferred by investors, the mainstream trading bond types in the inter-bank market are more influential in the generalized volatility spillover variance decomposition network of bond markets.

It should also be noted that Figure 3b is an undirected graph. In addition, the link between the nodes means that the influence is a two-way transmission. As is shown in Figure 3b, the biggest node is CDB, which is also the most traded variety of all in China’s bond market, and as the central node, it is linked with another two policy banks: ADB and IEB. CTBAAA and STNAAA are also important nodes thanks to their large trading volume.

The red nodes occupy the mainstream chain and are very closely connected, the trading volume of the inter-bank market is tremendous, and the weight of its main trading varieties is extremely significant.

After the analysis of Figure 3, the result of the MST complex network can be clearly seen in Table 4, corresponding with Figure 3.

It is worth mentioning these new emerging indicators, especially the centrality. In a holistic view of the results in Table 4, CDB is undeniably the most important node in the MST complex network of China’s bond market, due to its dominant position in relation to all four indicator rankings, including degree, closeness centrality, betweenness centrality and eigen centrality, showing that CDB not has only the most edges, but also the most influential neighbor nodes and the minimum average distance, proving that it is actually the central node of this complex network, thus demonstrating the systemic significance of China Development Bank. ADB is second to CDB, having the second highest centrality indicator performance, with a degree of 3. From the positions of CDB and ADB, it can clearly be seen that the bonds issued by China’s policy banks have great influence in the bond market, and are also frequently traded in the inter-bank market. However, the third highest ranking bond according to all the indicators is a bond issued by the left policy bank named Export-Import Bank of China (short as IEB); the main reason for this might be that the trading volume of IEB is slightly smaller than CDB and ADB. STN occupies second place in degree ranking and third place in all the centrality indicator rankings. This might also be thanks to the fact that medium- and short-term notes (short as STN) exist as a type of bond, akin to a bridge between short-term bonds and long-term bonds. The ranking of the other bonds are mainly positively related to the liquidity and trading volume, in line with common sense regarding bond trading activities, and tin varieties traded in the inter-bank market have obvious privilege.

Combined with Figure 3 and Table 4, from the perspective of the importance of bonds issued by financial institutions, the ranking is as follows: China Development Bank (bond) > Agricultural Development Bank (bond) ≈ bonds issued by banks and short- and medium-term notes with a rating of AAA (one of the most important inter-bank market trading type) >= Export-Import Bank (bond). In terms of institutional systemic importance, the regulator and policymaker must guarantee the capital adequacy ratio of these core institutions and the requirements of the Basel III, which also called «International Convergence of Capital Measurement and Capital Standards».

As the most influential type of credit bond which could also be traded in the inter-bank bond market, urban investment bonds have their own special advantages called “urban investment beliefs” and a large transaction volume. These “beliefs” stem from the implicit guarantee from the local governments, and urban investment bonds are usually invested in government-related construction projects. To prevent systemic financial risks, the default risk of urban investment bonds needs to be carefully considered in the position of systemic importance, especially there are already a few urban investment bonds which have technically defaulted recently.

### 5.2. Dynamic Spillover Effect Analysis

It is generally accepted that the spillover effect will change over time, and the relevance of different markets may intensify or decrease under uncertain conditions and unexpected shocks. In other words, the full-sample spillover index mentioned in the previous section is static, and might ignore the impact brought about by various political and financial events, such as the European sovereign debt crisis in 2009 and the violent fluctuations and crash of the Chinese stock market in 2015. The impact of these events during the sample period will exacerbate the spillover effect between different participants in the market and the risk crossing into different markets.

Taking the possibilities mentioned above into account, it seems that any static model with a single fixed parameter cannot reflect the evolution of the entire interval of the sample over time. Therefore, this research uses the sliding window method to study the time-varying spillover effects of different bonds, and through the total spillover index corresponding to the time series evaluates the degree and main characteristics of dynamic spillover effects. From the perspective of econometrics, the forecast step and the accuracy are negatively correlated. Perron and Qu’s research [51], which identified the structural change points of the dynamic spillover index series by the unit root test, used a 200-day sliding window and a 12-day forecast step. Taking the limitation of the number of observation points in the entire sample into account, retaining more instant spillover effect information in the bond market, this study uses a 150-day sliding window and a time-varying model with a 5-day forecast step to construct a dynamic volatility spillover index.

**Figure 4:**
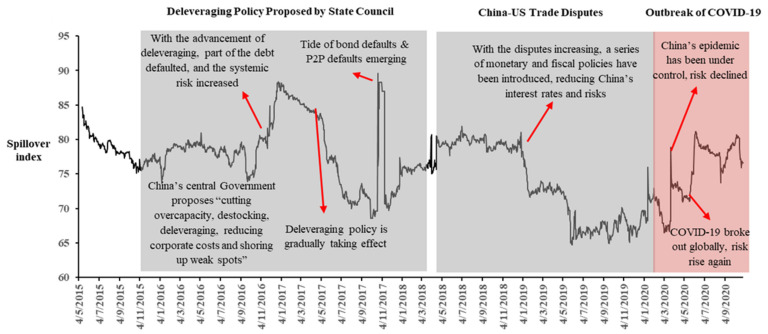
The dynamic evolution and trend of spillover effect of China’s bond market, including description of shock events (15 December 2014–28 October 2020).

As Figure 4 depicts, the volatility of China’s bond risk spillover index from 2015 to 2020 can be divided into three stages: (1) deleveraging policy proposed by state council, (2) China–US trade disputes, and (3) outbreak of COVID-19. From the perspective of bond systemic risks represented by changes in dynamic volatility spillover variance decomposition coefficients, with the expansion of China’s bond market and the continuous improvement of regulations issued by the governments, as well as with the gradual decline of real interest rates, the overall systemic risk trend falls slightly, and it is undeniable that the “Deleveraging policy” proposed by State Council played an important role in this process. However, it can also be seen that the shock caused by the rapid spread of coronavirus (COVID-19) has had dramatic impacts on financial markets all over the world [52]. It has created an unprecedented level of risk, causing investors to suffer significant loses in a very short period of time. With the tight liquidity and related expectations of the financial market, the systemic risk of China’s bond market has actually increased, while the risk has slightly decreased with the government’s macro-control after a short time, while finally, with the overall economic expectations moving toward pessimism and the rise of the global epidemic, the systemic risks have demonstrated a raising trend again. These pronounced and persistent impacts of the coronavirus pandemic upon Chinese financial markets correspond with recent research [53,54]. At the same time, systemic risks show a certain seasonal effect, which is related to the characteristics of liquidity changes in the bond market itself.

**Figure 5:**
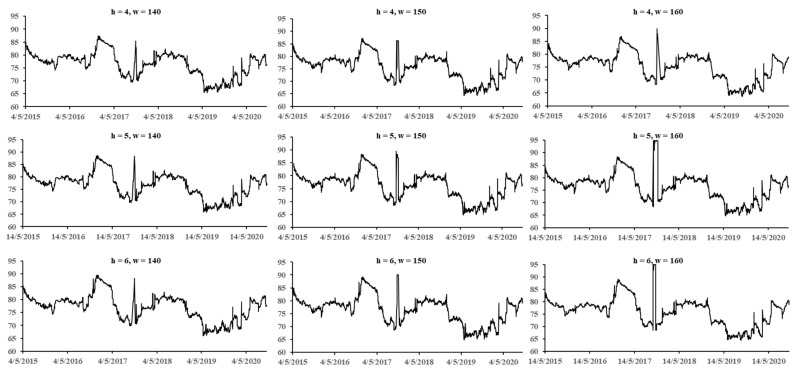
Robustness result of dynamic spillover effect of China’s bond market, with forecast step = 4, 5, 6 days and time window = 140, 150, 160 days.

To test the robustness of the results of dynamic spillover effect analysis, several hyperparameters were applied for comparison: forecast horizons (i.e., h = 4, 5, 6 days) and rolling window width (i.e., w = 140, 150, 160 days). In Figure 5, it is shown that the spillover index of China’s bond market follows a similar volatility pattern for the different values of h and w, concluding that the results of the study are robust regarding consistency.

In addition, to make the results more convincing and robust, possible future research could be expanded into several areas, such as the robustness of other methods or conducting dynamic analysis of networks [55].

**Figure 6:**
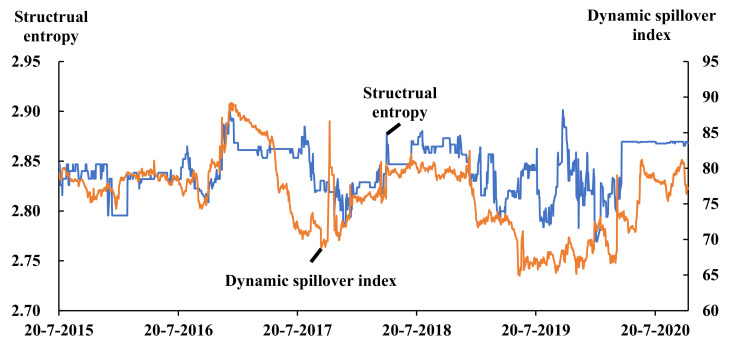
The complexity of the network of China’s bond market and the dynamic spillover effect of China’s bond market, represented by the structural entropy and the spillover index, respectively.

To verify the analysis of the dynamic spillover effect of China’s bond market and to discover the complexity of the bond market as a complex system, we calculated the structural entropy in a moving time window, which has a length of 150 days with a step size of 1 day, meaning that 1326 observations of structural entropy were generated. It is worth mentioning that from Figure 3a and Table 3, we can see that the complex network is an all-connected network, which means that the structural entropy of the network would be constant, making it worthless for the study, so the authors decided to cut some edges of weak connections, standing for the low spillover effect, to calculate the structural entropy. After observing the spillover coefficient distribution in Table 3, combining the analysis of the data correlation coefficient distribution and multiple adjustment attempts, it was found that the empirical result is relatively clear when the threshold is set to 5 percent, so the threshold was set at 5 percent, which means the edge between node *i* and *j* would exist only if $w_{ij}$ in Formula (8) ≥ 5 percent, otherwise the edge would be cut off. After the calculation from Formula (11), the result is shown as Figure 6:

In this study, the node number of the complex network is always 23; that is, the increase in system complexity caused by the increase in the number of the nodes, which is a very common phenomenon as an interference, does not appear in the research [21]. From Figure 6, we can see that the structural entropy and the dynamic spillover index has the similar pattern of the fluctuation. The correlation between the structural entropy and the spillover index is 0.451, and the *p*-value of the correlation is 0.0000, which means that the complexity of the complex network is statistically significantly positively correlated with the spillover index, and the result is statistically reliable. From this result, it can be concluded that, with the strengthening of the node connections within the network, the structural entropy, standing for the complexity of the complex network, will rise, while the systemic risk of China’s bond market also increases. The structural entropy could also be used as an effective indicator to measure the systemic risk, especially in the financial systems, which means that structural entropy could be used as a useful risk indicator to guide investment activities and show investors changes in the financial market or risk in their investment portfolio. In the meantime, structural entropy could also be an important reference for financial market regulators to assess financial risks.

## 6. Discussion and Conclusions

In this research, we document the evolution of the dynamics of risk spillover networks based on the complex network of China’s bond market by using daily interest rate data of representative bond categories in the Chinese bond market. At the very beginning, we construct an innovative correlation complex network and an MST network of China’s bond market, and these studies conduct a dynamic analysis based on a generalized vector autoregressive model, for which the volatility spillover variance decomposition method has been used to construct a complex network, and we adopt the minimum spanning tree method to analyze the clear transmission path of each bond’s interest rate and its volatility. Here are the main conclusions:

Firstly, it has been concluded that the importance of each bond type in the Chinese bond market is positively correlated with the main characteristics of bond-like liquidity, transaction volume, and credit rating, etc.

Secondly, the inter-bank market is the most important market in China’s entire bond market, without any doubt. In addition, interest rate bonds, commercial bank bonds and urban investment bonds are important bond types with systemic importance, which can be clearly seen in the complex networks constructed by the static spillover index.

Thirdly, from Figure 4 and Figure 6, we can see that the long-term trend of the dynamic spillover index of China’s bond market falls in line with the pace of interest rate adjustments, while several macro events such as the COVID-19 epidemic could bring instant shock which might cause systemic risk in China’s bond market, and furthermore, systemic risks show a certain seasonal effect. To hold the bottom line of preventing financial systemic risks in China’s bond market, standard management, strict supervision, and timely regulation of the bond markets are required, and the structural entropy, as a useful indicator for the complex network of the financial system, also should be used in risk management and monitoring.

Based on the conclusions above, corresponding policy recommendations can be put forward:

First, it is recommended to strengthen the monitoring and early warning systems of the fluctuations of China’s bond market, especially for the inter-bank market. The inter-bank market has the characteristics of large transaction volumes, a variety of bond trading types, and an upstream position of the capital. Drastic fluctuations in the inter-bank market will be transmitted to the downstream financial market, and even the real economy would be affected. In order to serve the real economy better, the supervision and regulation of the inter-bank market should be one of the top priorities in the work of policymakers, implementers and regulators.

Second, for issuers of the bonds with systemic importance in the volatility spillover network, the government and regulatory agencies of China should regard them as systemically important institutions in the network of bond market participants such as bond traders and market makers, and they need to propose higher standards of capital adequacy ratio and other requirements, to ensure that it can fully comply with the requirements of the Basel Agreement.

Third, investors in China’s bond market need to pay more attention to the credit rating and liquidity of bonds. Moreover, they need to pay more attention to bonds that are traded in the inter-bank market, such as commercial bank bonds and urban investment bonds.

The above conclusions have profound policy-guiding significance. On the one hand, China’s policymakers could comprehensively consider financial decisions related to China’s bond market from a networked perspective, thereby optimizing relevant decisions; on the other hand, from the standpoint of the China’s government, identifying economic areas which are closely related to China’s bonds market and financial institutes which have systemic importance in a timely manner has great forward-looking guiding significance for China’s government’s goal of maintaining the bottom line of preventing systemic financial risks.

## Figures and Tables

**Table 1:** The interest data of bonds chosen by this study.

Bond Type (Remaining Maturity: 1 Year If Not Mentioned)	Details and Description	Abbreviation
Commercial Banks Bonds	Commercial Banks Bonds (Rating: AAA)	BANKAAA
Corporate Bonds	Corporate Bonds (Rating: AAA)	CORPAAA
	Corporate Bonds (Rating: AA+)	CORPAAP
	Corporate Bonds (Rating: AA)	CORPAA
Treasury	China’s Treasury	TREASURY
Financial Bonds of Policy Banks	China’s National Development Bond	CDB
	China’s Agricultural Development Bond	ADB
	China’s Export-Import Bank Bond	IEB
Short- and Medium-Term Notes	Short and Medium Term Notes (Rating: AAA)	STNAAA
	Short and Medium Term Notes (Rating: AA+)	STNAAP
NCD (Interbank negotiable certificates of deposit)	Interbank negotiable certificates of deposit (Rating: AAA)	BANKIDCAAA
	Interbank negotiable certificates of deposit (Rating: AA+)	BANKIDCAAP
Consumer Financial Asset-backed Securities	Consumer Financial Asset-backed Securities (Rating: AAA)	CFABSAAA
	Consumer Financial Asset-backed Securities (Rating: AA+)	CFABSAAP
General Corporate Asset-backed Securities	General Corporate Asset-backed Securities (Rating: AAA)	ABSAAA
China’s Railway Bond	China’s Railway bond	RAILWAYB
Local Government Bond	Local Government Bond (Rating: AAA)	GOVAAA
Urban Investment Bond (Chengtou Bond)	Chengtou Bond (Rating: AAA)	CTBAAA
	Chengtou Bond (Rating: AA+)	CTBAAP
Credit Spread of State-owned Enterprises	Credit Spread of SOE	CSPREADSOE
R007 (remaining maturity: 7 days)	Seven-day repurchase rate	R007
DR007 (remaining maturity: 7 days)	Seven-day repurchase rate between deposit institutions	DR007
SHIBOR	Shanghai Interbank Offered Rate	SHIBOR1Y

**Table 2:** The statistic feature of the bond market’s interest data.

Series	T-Stats	Mean	Std Error	Minimum	Maximum	Skewness	Kurtosis	Stationary
BANKAAA	−30.887	−0.0010	0.0415	−0.4156	0.2500	−1.3653	17.5455	1st difference
CORPAAA	−24.226	−0.0011	0.0368	−0.2350	0.2292	−0.0761	6.7735	1st difference
CORPAAP	−24.188	−0.0014	0.0377	−0.2350	0.2292	−0.0117	5.5615	1st difference
CORPAA	−25.260	−0.0016	0.0391	−0.2350	0.2292	0.1380	5.0190	1st difference
TREASURY	−26.710	−0.0004	0.0335	−0.3100	0.3500	0.1439	20.1279	1st difference
CDB	−26.968	−0.0009	0.0400	−0.3529	0.2697	−0.7940	12.5871	1st difference
ADB	−23.285	−0.0009	0.0420	−0.2972	0.3673	−0.1849	13.8221	1st difference
IEB	−21.325	−0.0009	0.0403	−0.2972	0.3673	0.0009	13.2908	1st difference
STNAAA	−22.061	−0.0011	0.0368	−0.2350	0.2292	−0.0905	6.6818	1st difference
STNAAP	−22.915	−0.0014	0.0379	−0.2350	0.2292	−0.0455	5.4097	1st difference
BANKIDCAAA	−25.728	−0.0011	0.0414	−0.4130	0.2500	−1.3529	17.5522	1st difference
BANKIDCAAP	−25.493	−0.0010	0.0416	−0.4030	0.2500	−1.1148	15.1680	1st difference
CFABSAAA	−26.411	−0.0013	0.0421	−0.4088	0.2574	−0.7591	11.7487	1st difference
CFABSAAP	−26.406	−0.0015	0.0446	−0.4359	0.2744	−0.7592	12.0034	1st difference
ABSAAA	−26.326	−0.0013	0.0403	−0.3970	0.2500	−0.8645	12.9141	1st difference
RAILWAYB	−22.713	−0.0010	0.0366	−0.2325	0.2159	−0.2254	5.7954	1st difference
GOVAAA	−24.470	−0.0005	0.0323	−0.2486	0.3500	0.6673	17.5992	1st difference
CTBAAA	−24.195	−0.0012	0.0363	−0.2531	0.2889	0.0380	9.2296	1st difference
CTBAAP	−24.395	−0.0015	0.0366	−0.2531	0.2889	0.1237	8.0929	1st difference
CSPREADSOE	−60.674	−0.0002	0.1163	−0.8625	0.8883	−0.1068	33.1377	yes
R007	−30.752	−0.0004	0.2417	−2.3025	1.8934	−0.7548	20.5438	1st difference
DR007	−30.809	−0.0007	0.1125	−0.6976	1.3919	1.1032	22.9221	1st difference
SHIBOR1Y	−11.817	−0.0010	0.0153	−0.1740	0.0850	−2.8415	24.3002	1st difference

**Table 3:** The static spillover effect of the full sample.

	BANK AAA	CORP AAA	CORP AAP	CORP AA	TREASURY	CDB	ADB	IEB	STN AAA	STN AAP	BANKIDC AAA	BANKIDC AAP	CFABS AAA	CFABS AAP	ABS AAA	RAILWAYB	GOV AAA	CTB AAA	CTB AAP	CSPREADSOE	R007	DR007	SHIBOR1Y	From others
BANKAAA	26.5	12.1	11.4	9.5	3.2	7.4	0.6	0.6	0.4	0.4	0.3	0.3	0.2	0.2	0.2	0.4	3.1	11.1	9.9	0.1	0.9	0.2	1.1	73.5
CORPAAA	8.7	19.6	17.5	14.3	2.8	6	0.5	0.5	0.3	0.4	0.3	0.3	0.1	0.1	0.2	0.3	2.5	13.1	11.4	0	0.6	0.1	0.3	80.4
CORPAAP	8.2	17.6	19.2	15.2	2.7	5.8	0.6	0.6	0.3	0.4	0.3	0.4	0.2	0.2	0.2	0.3	2.6	12.3	11.4	0	0.7	0.2	0.4	80.8
CORPAA	7.7	16.1	17	21.2	2.4	5.3	0.4	0.4	0.2	0.3	0.2	0.3	0.1	0.1	0.1	0.2	2.3	12.3	11.7	0	0.7	0.2	0.3	78.8
TREASURY	3.4	3.7	3.3	2.6	36.7	8.3	0.8	0.9	0.3	0.4	0.2	0.4	0.1	0.1	0.1	0.2	29.1	3.9	3.4	0.4	0.3	0.3	1.1	63.3
CDB	7.5	7.5	6.9	5.7	6.8	35	1.1	1	0.8	1	0.5	0.6	0.3	0.3	0.3	0.7	5.7	8	6.8	0.8	1.8	0.4	0.3	65
ADB	0.4	0.2	0.2	0.1	0.1	0.2	25	20.4	7.3	6.8	6.8	6.7	5.7	5.6	6.1	7.2	0.1	0.2	0.2	0.1	0.4	0.1	0.1	75
IEB	0.3	0.2	0.2	0.1	0.1	0.1	20.1	25.5	7.3	6.7	7	6.9	5.7	5.7	6.3	7	0.1	0.2	0.2	0	0.2	0.1	0.1	74.5
STNAAA	0.2	0.1	0.1	0	0	0.1	6	6.1	16.9	15.5	8.3	8.2	7.8	7.8	8.2	14.3	0	0.1	0	0	0.1	0	0.2	83.1
STNAAP	0.2	0.1	0.1	0.1	0	0.1	6.1	6.2	16.2	17.2	8	7.9	7.7	7.7	8.1	13.8	0	0.1	0.1	0	0.1	0	0.2	82.8
BANKIDCAAA	0.2	0.1	0.1	0	0	0	5.4	5.7	8	7.3	15.6	14.6	11.4	11.4	12.1	7.7	0	0.1	0.1	0	0.2	0	0.1	84.4
BANKIDCAAP	0.2	0.1	0.1	0.1	0	0.1	5.4	5.7	8.1	7.5	14.8	15.8	11.1	11.1	11.7	7.7	0	0.1	0.1	0	0.2	0	0.1	84.2
CFABSAAA	0.1	0	0	0	0	0	4.7	4.7	7.7	7.2	11.4	11	15.3	15.2	14.5	7.7	0	0.1	0.1	0	0.2	0	0	84.7
CFABSAAP	0.1	0	0	0	0	0	4.7	4.7	7.6	7.2	11.5	11	15.2	15.3	14.6	7.7	0	0.1	0.1	0	0.2	0	0	84.7
ABSAAA	0.1	0	0	0	0	0	4.8	4.9	7.8	7.3	11.9	11.3	14.2	14.2	15	7.8	0	0.1	0.1	0	0.2	0	0	85
RAILWAYB	0.2	0.1	0.1	0	0	0.1	6.1	6.2	14.8	13.5	8.5	8.3	8.2	8.2	8.6	17	0	0	0	0	0.1	0	0.1	83
GOVAAA	3.4	3.8	3.6	3	29.1	7.2	0.7	0.7	0.2	0.3	0.2	0.3	0.1	0.1	0.1	0.2	36.7	4.2	3.6	0.3	0.2	0.2	1.6	63.3
CTBAAA	8.3	13.6	12.6	10.9	2.9	6.6	0.5	0.6	0.4	0.5	0.5	0.6	0.3	0.3	0.3	0.4	2.7	19.7	17.2	0	0.4	0.1	0.5	80.3
CTBAAP	7.8	12.7	12.5	11	2.9	6.4	0.6	0.6	0.4	0.6	0.5	0.6	0.3	0.3	0.4	0.4	2.8	18.1	19.8	0	0.4	0.3	0.5	80.2
CSPREADSOE	0.6	0.5	0.5	0.5	0.6	1.9	0.6	0.1	0.4	0.4	0.1	0.1	0.2	0.2	0.2	0.1	0.5	0.4	0.4	90.3	0.7	0.2	0.4	9.7
R007	2.4	2.2	2.4	2.2	0.5	3.7	0.9	0.8	0.4	0.5	0.8	1	0.7	0.7	0.7	0.6	0.4	1.4	1.3	0.1	60	16.7	0.1	40.3
DR007	1.1	0.5	0.9	1	0.8	1.9	0.5	0.5	0.2	0.4	0.2	0.4	0.1	0.1	0.2	0.3	0.5	0.6	0.9	0.2	21	67.4	0.4	32.6
SHIBOR1Y	8.1	4.7	4.3	3.3	3.8	7	0.4	0.4	1.1	1	0.3	0.3	0.1	0.1	0.1	0.8	3.4	5.9	4.8	0.6	0.1	0.8	48.7	51.3
Contribution to others	69	96	93.8	79.7	58.9	68.2	71.5	72.2	90.6	85.3	92.7	91.4	89.8	89.9	93.4	85.9	56	92.3	83.8	3.1	30	20.2	8	
Contribution including own	95.4	115.6	113	100.9	95.6	103.2	96.4	97.7	107.5	102.4	108.2	107.3	105.1	105.1	108.4	102.9	92.7	111.9	103.5	93.4	89	87.6	56.7	

**Table 4:** The statistical features of the MST complex network of China’s bond market.

Bond	Degree	Closeness Centrality	Betweenness Centrality	Eigen Centrality
CDB	5	0.38	0.72	1.00
ADB	3	0.36	0.54	0.73
STNAAA	4	0.32	0.50	0.65
CTBAAA	3	0.32	0.39	0.63
TREASURY	2	0.29	0.17	0.47
R007	2	0.29	0.09	0.44
BANKIDCAAA	3	0.27	0.32	0.44
CORPAAA	3	0.26	0.26	0.41
CSPREADSOE	1	0.28	0.00	0.37
IEB	1	0.27	0.00	0.27
STNAAP	1	0.25	0.00	0.25
RAILWAYB	1	0.25	0.00	0.25
ABSAAA	2	0.22	0.17	0.25
CTBAAP	1	0.24	0.00	0.24
GOVAAA	2	0.23	0.09	0.22
CORPAAP	2	0.21	0.09	0.21
BANKIDCAAP	1	0.22	0.00	0.18
DR007	1	0.22	0.00	0.17
BANKAAA	1	0.21	0.00	0.17
CFABSAAP	2	0.19	0.09	0.15
CORPAA	1	0.18	0.00	0.09
SHIBOR1Y	1	0.19	0.00	0.09
CFABSAAA	1	0.16	0.00	0.07

## Data Availability

All the data supporting reported results could be found in WIND.
